# The role of genetics in coronary artery bypass surgery patients under 30 years of age

**DOI:** 10.5830/CVJA-2016-042

**Published:** 2017

**Authors:** Sabit Sarikaya, Ebuzer Aydin, Yucel Ozen, Tanıl Ozer, Kaan Kirali, Murat Bulent Rabus

**Affiliations:** Cardiovascular Surgery Department, Kartal Kosuyolu Research and Education Hospital, Istanbul, Turkey; Cardiovascular Surgery Department, Kartal Kosuyolu Research and Education Hospital, Istanbul, Turkey; Cardiovascular Surgery Department, Kartal Kosuyolu Research and Education Hospital, Istanbul, Turkey; Cardiovascular Surgery Department, Kartal Kosuyolu Research and Education Hospital, Istanbul, Turkey; Cardiovascular Surgery Department, Kartal Kosuyolu Research and Education Hospital, Istanbul, Turkey; Cardiovascular Surgery Department, Kartal Kosuyolu Research and Education Hospital, Istanbul, Turkey

**Keywords:** coronary artery bypass graft (CABG),, plasminogen activator inhibitor (PAI)-1,, glycoprotein IIIa (GpIIIa),, methylenetetrahydrofolate reductase (MTHFR),, young patient

## Abstract

**Aim:**

We undertook genetic assessment of coronary artery disease (CAD) in 20 patients aged 30 years or less undergoing coronary artery bypass grafting (CABG) surgery, to investigate the prognostic value of pre-defined genes.

**Methods:**

Twenty patients, who underwent CABG surgery between December 2001 and May 2013, were retrospectively analysed to find out the role their genetic make-up played in their disease. We used three genetic diagnostic tests, the plasminogen activator inhibitor (PAI)-1 gene, the A1/A2 polymorphism of glycoprotein IIIa (GpIIIa) gene, and common polymorphisms of the methylenetetrahydrofolate reductase (MTHFR) gene.

**Results:**

The mean age of patients was 26.35 ± 3.51 (19–30) years, and 90% were male (n = 18). One patient had diabetes, three had hypertension, 11 (55%) had dyslipidaemia and 16 (80%) were smokers. Eight of the patients (40%) had left ventricular ejection fraction (LVEF) < 50%, and functional capacity was poor in only two (10%) patients (NYHA III– IV). Follow up was completed in all patients (100%). We found five homozygous and 11 heterozygous mutations in the MTHFR gene, which predisposes individuals to coronary artery disease or deep-vein thrombosis. Eight patients were found to have a GpIIIa gene polymorphism, which is associated with increased risk of myocardial infarction (MI). Fifteen patients had a polymorphism in the promoter region of the PAI-1 gene, which is a major inhibitor of the fibrinolytic system.

**Conclusion:**

MTHFR C677T polymorphism, and GpIIIa and PAI-1 genes are risk factors for CAD. In young patients, genetic studies promise to revolutionise early diagnosis, treatment and prevention of CAD and MI.

## Aim

Although atherosclerotic cardiovascular disease is known as a disease of the elderly, an advanced stage of disease requiring intervention may be also encountered in younger people. Increased experience in interventional procedures and technological improvements enable us to treat young individuals who have the disease, thereby avoiding a deterioration in their quality of life.

Although percutaneous interventional treatment is the first choice in appropriate cases, surgical treatment may also be selected as the first choice in young adult patients with advanced and multi-vessel disease, and is accompanied by early mobilisation and decreased duration of hospitalisation.^1^,^2^ When coronary artery bypass graft surgery (CABG) is indicated in young patients, the choice of operative technique, conduit selection and postoperative risk-factor modification should be carefully considered to prolong the graft patency and to avoid premature death.^3^

Although cardiovascular genetic studies lag behind genetic studies on other diseases, many advances have been made recently. Polymorphisms are gene variations that have only modest effects on the function of coded proteins or enzymes. However, they are common and can act as a risk factor together with the presence of environmental risk factors (cholesterol, stress, tobacco).

Current advances in molecular biology make it possible to detect numerous polymorphisms that might have a detrimental effect on vascular pathology, hence the hypothesis that multiple polymorphisms in the presence of environmental factors could act synergistically in the pathogenesis of atherosclerosis and coronary artery disease (CAD), which are typically polygenic and multifactorial diseases. There are many genetic diagnostic tests and we selected three for this study, the plasminogen activator inhibitor (PAI)-1 gene, the A1/A2 polymorphism of glycoprotein IIIa (GpIIIa) gene, and a common polymorphism of the methylenetetrahydrofolate reductase (MTHFR) gene, the thermolabile C677T.

## Methods

CABG was performed either isolated or concomitantly with other cardiac surgical interventions on 16 281 patients between December 2001 and May 2013 at our clinic. Twenty patients who were 30 years of age or older were included in this retrospective study. Pre-, intra- and post-operative characteristics of the patients were evaluated. The study protocol was approved by the Institutional Ethics Committee, and all patients gave consent to participate in the study. Medical data of the patients were reviewed, and patients were also called on to gather information about their recent health status.

Cardiac operations were performed on a beating heart or by providing cardiac arrest under cardiopulmonary bypass. Various techniques were used for cardiac stabilisation in operations performed on a beating heart. Isothermic, hyperkalaemic blood cardioplegia was used via the antegrade route and/or retrogradely in order to provide cardiac arrest. Grafts were prepared perioperatively as autogenous grafts. The left (LIMA) and right internal mamarian artery (RIMA) and the radial artery were prepared as arterial grafts, whereas the great sapheneous vein was used as a venous graft.

The total genomic DNA in the patients’ peripheral whole blood samples was isolated using GenXtract solution according to the manufacturer (Vienna-Lab Diagnostic GmbH). Afterwards, target DNA regions were amplified with multiflex polymerase chain reaction (PCR) using biotinylated primers, and amplified products were controlled with 3% agorose gel. After observing the amplicons belonging to the test strip containing immobilised allele-specific nucleotide probes on nitrocellulose membrane using CVD strip assay (Vienna-Lab Diagnostic GmbH), a hybridisation process was performed with profiblot T48 (Tecan) hybridisation instruments. Bound biotinylated sequences were detected using streptavidin-alkaline phosphatese and colour substrates.

## Statistical analysis

Statistical analysis was performed using SPSS software version 12.0 (SPSS Inc, Chicago, IL, USA). Numerical parameters are expressed as mean, median and standard deviations.

## Results

Mean age of the patients was 26.35 ± 3.51 (19–30) years, and 90% were male (n = 18) (Table 1). More than half of the patients had normal body mass index (BMI), and all had an urban lifestyle. Detailed medical information was gathered about familial and/or sporadic diseases, personal history, habits and family history (Table 2). One patient had diabetes, three had hypertension, 11 (55%) had dyslipidaemia, and 16 (80%) were smokers. Eight of the patients (40%) had a left ventricular ejection fraction (LVEF) < 50%, and functional capacity was poor in only two (10%) patients (NYHA III–IV).

**Table 1:** Demographic characteristics fo the patients

Age (mean ± SD), years	26.35 ± 3.51 (19–30)
Gender	
Male, n (%)	18 (90)
Female, n (%)	2 (10)
BMI, kg/m2, n (%)	
> 30	3 (15)
20–30	14 (70)
< 20	3 (15)
Geographical region	
Rural, n (%)	0 (0)
Urban, n (%)	20 (100)

SD: standard deviation; BMI: body mass index.

**Table 2:** Basline characterristics of patients

Patient characteristics	Number of patients	Percent
Hypertension	3	15
Diabetes	1	5
Hyperlipidaemia	11	55
Familial	5	25
Vasculitis		
Takayasu arteritis	2	10
Carotid artery disease	2	10
Positive family history	7	35
Smoking	16	80
Alcohol use	3	15
Echocardiography		
LVEF > 50%	12	60
LVEF < 50%	8	40
Valvular pathology	4	20
Coronary angiography		
Single-vessel disease	10	50
Dual-vessel disease	3	15
Multi-vessel disease	7	35
NYHA		
I–II	18	90
III–IV	2	10
Previous MI	6	30
Previous interventions		
CABG	0	0
PTCA/stent	2	10
Renal insufficiency	0	0
Chronic pulmonary diseases	1	5

LVEF: left ventricular ejection fraction; NYHA: NewYork Heart Association; MI: myocardial infarction; CABG: coranary artery bypass grafting; PTCA: percutaneous transluminal coranary angioplasty.

The stents of the two patients who had coronary angioplasty and were operated on for stent re-stenosis, had been implanted one and five months earlier due to acute myocardial infarction. Isolated CABG was performed in 19 patients, whereas right CABG was performed concomittantly with aortoplasty in only one patient due to aortic stenosis (Table 3). Cardiac stabilisation without perfusion, using various techniques of cardiac arrest (off-pump CABG), was performed in seven out of 11 patients with single-vessel bypass surgery and two out of three patients with dual-vessel bypass surgery. Other patients were operated on under cardiopulmonary bypass (CPB).

**Table 3:** Surgical information

Surgery	Number of patients
Isolated CABG	19
Off-pump	9
On-pump	11
Total perfusion time (mean ± SD), min	65.6 ± 24.6
Aortic cross-clamp time (mean ± SD), min	42 ± 22.3
Number of vessels with bypass surgery	
Single	11
Double	3
Multiple	6
Graft type	
LIMA	17
RIMA	1
Radial artery	1
Saphaneous vein	19

CABG: coranary artery bypass grafting; SD: standard deviation; LIMA: left internal mammary artery; RIMA: right internal mammary artery.

Isothermic hyperkalaemic blood cardioplegia was used for cardiac arrest. Mitral protection was provided by inducing moderate hypothermia and antegrade and/or the administration of blood cardioplegia. Bypass was performed with the LIMA flap to the left anterior descending artery (LAD) in all patients except three; two patients with peripheral artery disease due to vasculitis, and one who had right CABG surgery only. Bypass was performed with the RIMA on the right coronary artery (RCA) in one patient, and in another using a radial artery graft.

All except two patients were operated on electively (these patients had coronary artery dissection at postpartum, and acute myocardial infarction, and they had emergency surgery). According to the EUROscore, two patients were evaluated as moderate risk, and the others were low risk. Two patients with low ventricular function were placed in the intensive care unit (ICU) under inotropic support.

Patients were followed up routinely, and the extubation time was 7.4 ± 3.2 hours. Duration of ICU and hospital stay was 1.4 ± 0.9 and 5.6 ± 2.1 days, respectively. The medical treatment of two patients who were diagnosed with Takayasu’s arteritis and had severe extensive peripheral vasculopathy was continued as scheduled. Pre-operatively scheduled antihypertensive, antihyperlipidaemic and antidiabetic medical treatments were continued after surgery. Routine anticoagulant (acetylsalicyclic acid 150 mg/day) treatment was added on the first postoperative day. There were no hospital mortalities.

All patients were contacted using the contact information in their medical files, and all of the contacted patients were alive. All except one patient reported that they had no limitations in physical activities, or socio-economic and emotional aspects, and their quality of life was no different from that of the normal population. One patient said he was not actively working because he had respiratory distress and low functional capacity.

During control coronary angiography (CAG), one patient with dyspnoea, low functional capacity and moderate risk, who was operated on in April 2003, was diagnosed with obstructed saphenous vein grafts in December 2004, and it was observed that LIMA flow was slow and weak. Another patient, who had bypass surgery on the LAD and RCA in 2004, had coronary angiography performed in 2010 and a preliminary diagnosis of non-ST-segment MI. Angiography revealed that the grafts were patent, however there was critical stenosis in the circumflex artery (Cx), which had not undergone bypass surgery before. Angioplasty was performed on the Cx.

In another patient, a stent was implanted in January 2006, and LAD bypass was performed in June 2006 after stent stenosis. In the control CAG in October 2006, we observed that the LIMA was open. Control CAG in 2009 of an unsymptomatic patient who had had triple-vessel bypass in 2003 revealed that the grafts were patent.

Control CAG in 2007 of another patient with single-vessel bypass in 2003 revealed a patent graft. A patient with singlevessel bypass in 2004 also had a patent graft in 2009. Other patients who reported having no problems did not present to any hospital. Seven out of 20 followed-up patients said that they were still smoking the same number of cigarettes.

We found five homozygous and 11 heterozygous mutations in MTHFR, which predisposes individuals to CAD or deep-vein thrombosis (DVT). Eight patients were found to have a GpIIIa gene polymorphism, which is associated with increased risk of MI. Fifteen patients had a polymorphism in the promoter region of the PAI-1 gene, which is a major inhibitor of the fibrinolytic system.

## Discussion

Coronary artery disease is generally defined as a disease of advanced age because of its prevalence in older individuals. However it may also be encountered less frequently in younger people. Since they are active members of the national economy, it is clear that these young individuals should be treated in the most effective way. Successful treatment should involve uncomplicated and safe surgery, resulting in patient survival.^4^ Currently, criteria for successful treatment include not only patient survival but also duration of recovery and returning to an active life without any problems, as well the economic aspects of appropriate treatment options.^4^

Risk factors such as smoking and hyperlipidaemia are more commonly observed among young adults with severe coronary artery disease, whereas diseases such as hypertension and diabetes are more frequently observed in the elderly population.^5^,^6^ Cessation of smoking and improving dyslipidaemia may play a significant role in decreasing disease prevalence in early adulthood.^5^,^6^

CAD requiring CABG is common in middle-aged or elderly populations. In the current era, primary prevention includes identifying the genetic risk as well as optimising the modifiable risk factors. This is critical in this young population group before target-organ damage occurs.^7^

Selecting appropriate conduits for long-lasting graft patency is another important issue in these patients. The use of other arterial grafts such as the radial artery was proven to be superior to venous grafts for long-term patency, especially on the left coronary system.^8^ We observed some remarkably good early results in our series. Unfortunately, a more disappointing panorama can be observed when analysing their long-term evolution. Arteriosclerosis is a progressive disease and many patients in our series suffered its consequences during their follow up, as recurrent heart ischaemia, or major peripheral vascular complications, or both.

Genetic studies of CAD and MI are lagging behind genetic studies of other cardiovascular disorders. The major reason for the limited success in this field of genetics is that CAD and MI are complex diseases, believed to be caused by many genetic and environmental factors, and the interaction between these factors.^9^ Appropriate treatment and prevention of these diseases is difficult because they are multifactorial.

Many risk factors have been identified for CAD and MI, including smoking, advanced age, male gender, diabetes mellitus, high systolic blood pressure, personal history of angina pectoris, family history of CAD or MI, high-fat diet, infectious agents, obesity, increased plasma total and low-density lipoprotein (LDL) cholesterol levels, increased plasma triglyceride levels, and decreased plasma high-density lipoprotein (HDL) cholesterol levels.^10^-^12^ Among these factors, family history is one of the most significant independent risk factors for CAD and MI.^13^ This supports the hypothesis that genetic factors contribute to the development of CAD and MI, and we therefore used three genetic diagnostic tests to find a relationship between genetic make-up and heart disease.

Rare genetic defects that cause extremely high plasma homocysteine levels also cause CAD.^14^,^15^ It was therefore hypothesised that, even within the normal range of plasma homocysteine concentrations, higher levels may appreciably increase CAD risk.^15^ The enzyme methylene tetrahydrofolate reductase, encoded by the MTHFR gene, uses folate to metabolise and thereby remove homocysteine.^16^ The MTHFR C677T polymorphism is common (T-allele frequency 15–45%) in many populations and reduces enzyme efficiency.

We found five homozygous and 11 heterozygous mutations in the MTHFR gene and the others did not have mutations. Homozygosity for the C677T polymorphism of the MTHFR gene predisposes individuals to CAD or DVT.

The A1/A2 polymorphism of the GpIIIa gene caused by a T-to-C nucleotide substitution at position 1565, which is associated with the occurrence of the amino acid Leu →Pro variant at residue 33 of the mature protein,^17^ has been widely studied in cardiovascular diseases.^18^ These studies have shown that possession of an A2 allele increases the risk for MI,^19^,^20^ CAD,^21^ and restenosis after stent placement.22 Eight patients had a GpIIIa gene polymorphism in our study.

PAI-1 is a major inhibitor of the fibrinolytic system. This protein is under the control of the 4G/5G polymorphism of the PAI-1 gene, which is characterised by the presence of five guanine nucleotides in the promoter zone instead of four. Carriers of the 4G/5G allele would be more at risk for CAD.^22^-^25^ We had 15 patients who had this polymorphism.

This study was limited by the small sample size and ethnicity differences. Further exploration would be useful in future studies, for which a larger sample size is needed.

## Conclusion

This study demonstrates that the MTHFR C677T polymorphism, and GpIIIa and PAI-1 genes are risk factors for CAD. Considering that individuals who have homozygous mutations in the MTHFR gene are prone to CAD in early adulthood, it is possible that altered enzyme efficiency contributes to this vulnerability. Genetic studies promise to revolutionise early diagnosis, treatment and prevention of CAD and MI. A unique advantage for the management of cardiovascular disease is that a significant number of cases are potentially preventable. Early diagnosis by genetic testing will force lifestyle modifications in individuals with genetic risk factors, which alone or in combination with other therapeutic options may delay the onset of CAD or prevent MI.
